# Central Cord Syndrome in a Young Patient with Early Diffuse Idiopathic Skeletal Hyperostosis and Ossification of the Posterior Longitudinal Ligament after Minor Trauma: A Case Report and Review

**DOI:** 10.7759/cureus.284

**Published:** 2015-07-13

**Authors:** Michael Galgano, Lawrence S. Chin

**Affiliations:** 1 Neurosurgery, SUNY Upstate Medical University

**Keywords:** central cord syndrome, diffuse idiopathic skeletal hyperostosis, ossification of the posterior longitudinal ligament, spine trauma, spinal cord injury

## Abstract

This paper is a case report and literature review. The objective of this article is to address a rather unusual case of central cord syndrome in a patient with diffuse idiopathic skeletal hyperostosis and focal ossification of the posterior longitudinal ligament. We also discuss the mechanism of injury in central cord syndrome, as well as that specific to involvement of diffuse idiopathic skeletal hyperostosis (DISH) and ossification of the posterior longitudinal ligament (OPLL). This case took place at SUNY Upstate Medical University. We report a case of a 39-year-old male with early diffuse idiopathic skeletal hyperostosis and focal ossification of the posterior longitudinal ligament, presenting with central cord syndrome after minor trauma. The patient presented with tetraparesis, predominating with significant distal upper extremity weakness and hyperpathia. We performed a C3-6 decompressive laminectomy, with C2 pars screws, and C3-7 lateral mass screws. Since surgery, the patient has had a steady progressive improvement in neurological function and is currently ambulating with a good functional use of his upper extremities. An increased risk of spinal cord injury is a known risk in individuals with pre-existing spinal ankylosing. Few reports are present citing the contribution of focal OPLL with DISH in this age group within the cervical spine contributing to the central cord syndrome.

## Introduction

An increased risk of spinal cord injury is a known risk in individuals with pre-existing ankylosed spinal segments. We report a case in which a patient with early diffuse idiopathic skeletal hyperostosis (DISH) and focal ossification of the posterior longitudinal ligament (OPLL) presented with a central cord syndrome after minor trauma. Surgical intervention was subsequently undertaken. Although concurrent DISH and OPLL may be associated in generalized hyperostotic states, this relationship leading to central cord syndrome in a rather young patient is not reported in the literature.

## Case presentation

Informed verbal consent was obtained by the patient reported in this study.

**Figure 1:**
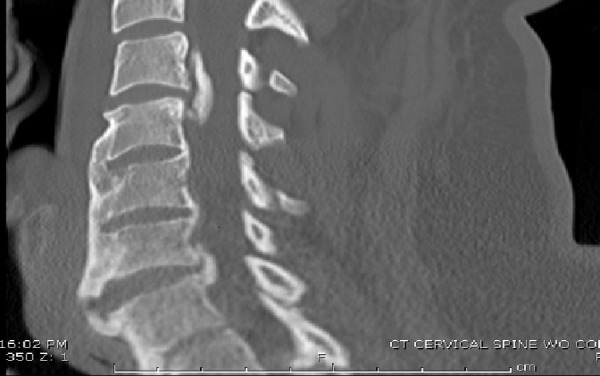
Preoperative sagittal cervical spine CT A focal ossified PLL, anterior autofusion from C4-T1, and a lucency through a C6-7 syndesmophyte

We report a case of a 39-year-old male with early diffuse idiopathic skeletal hyperostosis and focal ossification of the posterior longitudinal ligament presenting with central cord syndrome after minor trauma. The patient presented to our trauma center with tetraparesis, with upper extremity weakness predominating. He had significant hyperpathia with associated severe neck and back pain, in addition to urinary retention. Significant sensory deficits were present from his upper torso to the plantar regions of his feet bilaterally. Bilateral Hoffman’s sign was present, in addition to brisk lower extremity reflexes. Initial CT of the cervical spine revealed extensive ossification of the anterior longitudinal ligament. A focal region of the ossified posterior longitudinal ligament was prominent at the C3-4 level, in addition to anterior autofusion from C4-T1. A concerning lucency through a syndesmophyte at C6-7 was present (Figure 1).

**Figure 2:**
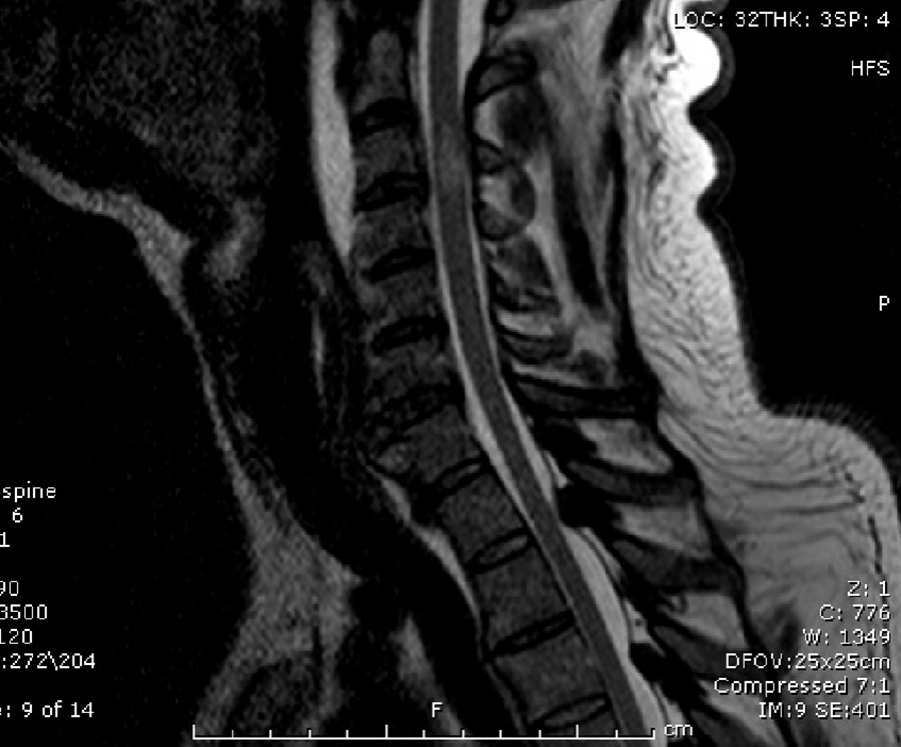
Preoperative sagittal cervical spine T2 MRI Cervical spine stenosis with a focal cord hyperintensity at the level of C3

MRI of the cervical spine revealed an acute spinal cord injury at C3-4, which was the level of the large posterior osteophyte complex (Figure 2).

**Figure 3:**
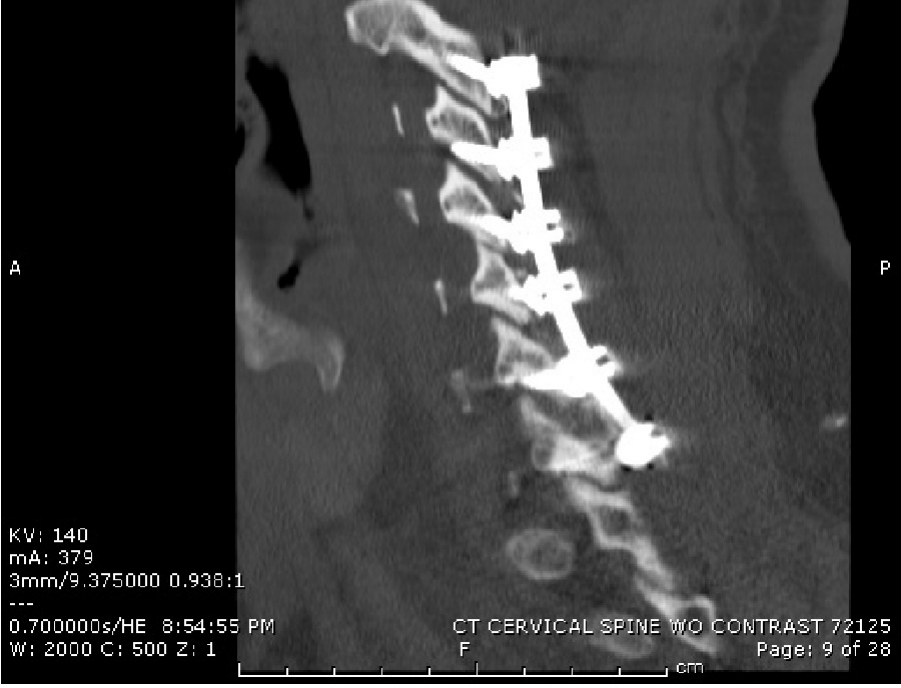
Postoperative sagittal cervical spine CT Intrumentation from C2-C7

No thoracic or lumbar spine fractures were noted. However, the patient did have scattered anterior thoracic osteophytic processes as well as very thickened ligamentum flavum at multiple regions posteriorly. Upon admission to the surgical intensive care unit, he was started on a methylprednisolone drip. The patient’s upper extremity function continued to decline, and we opted to intervene with surgical intervention. We performed a C3-6 decompressive laminectomy, with C2 pars screws, and C3-7 lateral mass screws. Intraoperatively, there was evident hypermobility between C3 and C4. Obvious distraction was visualized between the right C3 inferior articulating process and C4 superior articulating process. Postoperative CT and MRI scans showed adequate hardware placement (Figure 3).

Since surgery, the patient has had a steady progressive improvement in neurological function and is currently ambulating with good functional use of his upper extremities.

## Discussion

Diffuse idiopathic skeletal hyperostosis (DISH), also known as Forestier’s Disease, is a well-defined non-inflammatory disease with both spinal and peripheral manifestations [1]. When it manifests itself in the spine, anterior and lateral ossification of the vertebral bodies predominate. It is characterized by spinal longitudinal ligaments and entheses becoming progressively ossified, resulting in decreased mobility of the affected region [1]. Fractures of ankylosed spinal segments with increased overall mobility have been shown to be more unstable, as a result of ossified ligaments and surrounding tissue that also fractures. Unfortunately, the clinical outcomes in this patient population tend to be worse when compared to the general population in regards to spinal trauma [1].

Overall, about 70% of cases of DISH have involvement in the entire vertebral column [2]. In descending order of frequency, osteophytic processes are generally most prevalent in the thoracic, lumbar, and cervical regions. The majority of individuals with DISH are generally asymptomatic.

Central cord syndrome is a subtype of cervical spinal cord injury that is characterized by a disproportionate severity in loss of upper limb function as compared to lower limb function. Urinary retention, as well as varying levels of sensory deficits below the injury level, are also common [3]. This type of spinal cord injury is most common in older patients harboring some degree of cervical spondylosis suffering from a concomitant hyperextension injury. It is felt by many authors that the disparity in motor deficit seen in central cord syndrome rests on the premise that damage is to the most medial fibers of the lateral corticospinal tract, which serves the upper limb and hand.

To date, there have been few cited papers specifically reporting central cord syndrome after minor trauma in patients with early DISH and focal OPLL [4-5].^ ^The only other such cases reported in the literature were by Eser, et al. [4] as well as Razmi, et al. [5].

## Conclusions

It has been postulated that the longer an ankylosed spinal segment is at a particular fracture site, the worse the associated spinal cord injury tends to be. Multi-level ankylosed vertebral body segments may act as a long lever arm for traumatic forces to act upon and displace into neural elements, heightening the severity of the cord injury. In our particular case, there was anterior autofusion from C4-T1, coupled with a large focal area of an ossified posterior longitudinal ligament. These pathological features contributed to the patient’s spinal cord injury.

The case we have presented highlights the rare association of DISH and OPLL, leading to a central cord syndrome in a young patient after minor trauma.
